# Design and baseline characteristics of the Short bouTs of Exercise for Preschoolers (STEP) study

**DOI:** 10.1186/1471-2458-12-582

**Published:** 2012-08-01

**Authors:** Sofiya Alhassan, Ogechi Nwaokelemeh, Albert Mendoza, Sanyog Shitole, Melicia C Whitt-Glover, Antronette K Yancey

**Affiliations:** 1 Department of Kinesiology, University of Massachusetts Amherst, 110 Totman Building, 30 Eastman Lane, Amherst, MA 01003-9258, USA; 2 Gramercy Research Group, 500 West 4th Street, Suite 203, Winston-Salem, NC 27101, USA; 3 UCLA Kaiser Permanente Center for Health Equity, Fielding School of Public Health, University of California Los Angeles, Box 956900, 31-235 CHS, Los Angeles, CA 90095, USA

**Keywords:** Physical activity, Accelerometer, Preschoolers, Short-activity break

## Abstract

**Background:**

Most preschool centers provide two 30-min sessions of gross-motor/outdoor playtime per preschool day. Within this time frame, children accumulate most of their activity within the first 10 min. This paper describes the design and baseline participant characteristics of the Short bouTs of Exercise for Preschoolers (STEP) study. The STEP study is a cluster randomized controlled study designed to examine the effects of short bouts of structured physical activity (SBS-PA) implemented within the classroom setting as part of designated gross-motor playtime on during-school physical activity (PA) in preschoolers.

**Methods/Design:**

Ten preschool centers serving low-income families were randomized into SBS-PA versus unstructured PA (UPA). SBS-PA schools were asked to implement age-appropriate 10 min structured PA routines within the classroom setting, twice daily, followed by 20 min of usual unstructured playtime. UPA intervention consisted of 30 min of supervised unstructured free playtime twice daily. Interventions were implemented during the morning and afternoon designated gross-motor playtime for 30 min/session, five days/week for six months. Outcome measures were between group difference in during-preschool PA (accelerometers and direct observation) over six-months. Ten preschool centers, representing 34 classrooms and 315 children, enrolled in the study. The average age and BMI percentile for the participants was 4.1 ± 0.8 years and 69th percentile, respectively. Participants spent 74% and 6% of their preschool day engaged in sedentary and MVPA, respectively.

**Discussion:**

Results from the STEP intervention could provide evidence that a PA policy that exposes preschoolers to shorter bouts of structured PA throughout the preschool day could potentially increase preschoolers’ PA levels.

**Trial registration:**

Clinicaltrials.gov, NCT01588392

## Background

Physical activity (PA) is critical to the healthy development of preschool-age (2.9-5 year) children. It is recommended that preschoolers should accumulate at least 120 min (structured and unstructured) of PA per day [1]. Unfortunately, most preschoolers do not meet the current PA recommendation [2]. In general, children engage in approximately eight minutes of moderate to vigorous PA (MVPA) per hour of preschool attendance [3]. Thus, a child in full-day preschool (8 h) would engage in approximately 64 min of MVPA per day, or half of the recommendation [3]. Furthermore, it is unlikely that a child who attends a full-day of preschool would engage in additional MVPA outside of preschool [4]. In addition, while little research on the effects of prolonged sitting has been conducted in this population, the effects are unlikely to be less adverse than in older groups [5].

During a typical 30-min bout of gross motor playtime, direct observation and direct measures of PA (accelerometer data) indicate that preschoolers accumulate very few minutes of MVPA [6,7]. The accumulation of MVPA generally occurs during the first 10 min of play, with the remaining 20 min block spent in sedentary to very light intensity activities. The longer children participate in a given game or activity (structured or unstructured), the less activity they accumulate during the entire period of the activity [7]. Based on this evidence, it is possible that higher levels of MVPA could be attained by exposing children to shorter bouts of structured PA (e.g., ≤ 10 min) multiple times throughout the preschool day.

To date, PA policies in preschool centers have focused on changing the outdoor playtime environment and little (if any) attention has been given to changing the delivery of PA in the academic classroom. In one of the few studies that examined classroom PA, Trost et al. found that the incorporation of a PA curriculum during classroom circle time significantly affected PA levels of children attending half-day preschool [8]. Most low socioeconomic status (SES) children are in preschool for approximately eight hours per day, of which only one hour is spent engaged in gross motor playtime and the remaining seven hours (~86%) are spent in sedentary activity [9]. Currently, very little is known about the impact of short bouts of structured activity on preschoolers PA level during the school day. The Short bouTs of Exercise for Preschoolers (STEP) study was designed to examine the effects of short bouts of structured PA (SBS-PA) implemented within the classroom setting as part of designated gross-motor playtime on during-preschool total PA and sedentary time in low-SES preschoolers. This paper describes the design and baseline participant characteristics of the STEP study.

## Methods/Design

### General design

The STEP study is a cluster randomized, controlled six-month trial to examine the effects of a SBS-PA program implemented during the designated gross-motor playtime during the preschool day in preschool-aged children. Outcomes of interest include total preschool-day PA, percent of time spent in MVPA, and sedentary time during gross-motor playtime and total preschool-day. Preschool centers were randomly assigned to one of two groups: SBS-PA or traditional unstructured PA (UPA). All children within a given preschool center were allowed to participate in the assigned intervention. This study protocol is in compliance with the Declaration of Helsinki and has been reviewed and approved by a University of Massachusetts, Amherst human research review board.

### Participant recruitment and eligibility

A total of 15 preschool centers with a minimum of two full-day classrooms that serve low-SES areas in Springfield, Massachusetts area were approached to participate in STEP. Ten of the 15 preschool centers approached met the screening criteria and agreed to participate in the study. Reasons preschool centers declined participation included unwillingness to be randomized and inability to consistently provide 30 min of morning and afternoon gross-motor playtime per preschool day. Although not a requirement for eligibility, we assessed the existing PA policies for each of the 10 preschool centers enrolled in the study for randomization/stratification purposes. Existing PA policies were assessed using a modified version of the Environment and Policy Assessment and Observation Audit Tool (EPAO)[10]. The assessment included written PA policy, teacher training in PA and involvements with children during gross-motor playtime, and indoor and outdoor PA equipment.

Within each preschool center, between 25 – 30 children were individually recruited for the assessment portion of the study. Children were eligible for the assessment portion of the study if they: 1) attended one of the 10 participating preschool centers; 2) were between 2.9 – 5 years of age on the date of baseline assessment; 3) did not have a condition limiting their participation in MVPA (e.g., unable to participate in routine gross-motor playtime at preschool); 4) unable to wear the activity monitor, and 5) attend preschool full-time. Children that were excluded due to eligibility criteria or whose parents did not wish to sign the informed consent were still allowed to participate in the intervention protocol; however no assessment data were collected on them.

### Randomization

The STEP study utilized a cluster-randomized design because within a preschool center, classrooms sometimes share resources during gross motor playtime and thus, contamination between intervention arms would be unavoidable. Preschool centers were randomized into the treatment (SBS-PA) or control (UPA) group, stratified by preschool center size and existing PA policy. Randomization was performed using the Efron procedure [11].

### Interventions

Due to the PA requirements set forth by the Massachusetts State Department of Early Education & Care, all participating preschool centers in the current study were mandated to provide 60 minutes of gross-motor playtime per day. Typically preschool centers divide this playtime into two 30-min sessions—one in the morning and one in afternoon. For this study, both the treatment and control interventions were implemented during the designated morning and afternoon gross motor playtimes five days per week for six months.

### Treatment intervention

#### Theoretical framework

The study intervention was developed based on the social ecological model and the Meta-Volition Model (MVM) [12]. The MVM’s fundamental premise is that organizational practice and policy change is the key lever for population health behavior change. Of particular relevance for this project, the MVM posits that as classroom teachers experience the ease of implementation and benefits of the intervention for their own work (e.g., improved student attentiveness after PA breaks), this will reinforce and cement their adherence. These teachers will also comment favorably to other teachers, spurring engagement of additional classrooms and, ultimately, institutionalization within the center. MVM is built upon Social Cognitive Theory [13,14], and Diffusion of Innovations [15]. Social cognitive theory asserts that behavior develops, is altered, and maintained through the interplay of personal, behavioral, and environmental factors [16,17]. The Social Cognitive Theory construct most pertinent to the MVM is observational learning from role models [18] to increase self-efficacy and behavioral adoption [12,16,17,19]. Classroom teachers are thought to be the second largest category of role models among preschoolers, after family members. The involvement of classroom teachers accomplishes many things: it makes activity participation more appealing and culturally adaptable; it provides highly-valued but inexpensive incentives for participation that may help to reinforce the activities and may help generalize them to other settings; and it represents a more feasible and sustainable practice and policy change in low-resource schools than those requiring expensive equipment or expert personnel.

Additionally, these theoretical models include personal factors: the children’s and teachers’ interest and participation in SBS-PA; the children’s belief that they have the ability to perform behaviors that will secure desired outcomes (self-efficacy); and outcome expectation (the intervention will result in more fun for the students and more manageable students for the teachers). Preschoolers have developed some aspect of self-efficacy because at this age they know that their personal action can produce certain results. Behavioral factors include the knowledge and skills available and needed to perform the intervention; and the degree of competence attained in using these skills. The structured activities utilized in this intervention will be developmentally appropriate for the motor skills of preschool-aged children. Of the three determinants of behavior development, environmental factors (social or physical) will have the greatest relative influence on behavior in this intervention. With respect to this intervention, the social environment includes modeling by peers and adults (teachers). Preschoolers are able to pay attention and therefore peer and adult modeling of the intervention will enhance the children’s self-efficacy for learning and their ability to successfully perform the activities. The physical environment deals with the structurally integrated approach of the intervention (changes in routine, e.g., to plug in a DVD/CD or rearrange furniture). The intervention task has built-in reinforcement because the activities are designed to be fun.

#### Development of short bouts structured PA intervention: (tutti fruitti instant recess)

Tutti Fruitti Instant Recess (*TFIR*) was based on the Instant Recess® (*IR*) program, which has been utilized in different age populations [12,20-23]. *IR* is 10-min physical activity routines, set to music with simple movements that engage major muscle groups. *IR* is available in audio (CD) or audiovisual (DVD) format, and is utilized to incorporate active breaks into routine organizational settings (e.g., the workplace, church activities, classrooms) for adults and youth. The SBS-PA intervention (*TFIR*) consisted of 10-min routines recorded onto DVDs that featured music and movements appropriate for preschool-aged children. A total of 16 audiovisual DVDs were created—ten existing *IR* routines that were originally geared toward adults (http://www.toniyancey.com) were modified for the current study, and six new *TFIR* routines were created by the research staff. The *TFIR* program was created in collaboration with a physical education specialist. All routines were designed to be simple, easy to learn and included low- to moderate-impact aerobic movements. Within each routine, movements (choreographed to music set at 100–120 beats/min) started with lower body actions before the addition of upper body movements. Each 10-min *TFIR* routine consisted of a warm-up (1 min), moderate- intensity activity (8 min) and a cool-down (1 min). Warm-up and cool-down movements were set to the same musical tracks, while the 8-min moderate-intensity movements were set to two 4-min tracks. Each *TFIR* DVD was accompanied with a movement-by-movement verbal and photo guide to assist classroom teachers in implementing the intervention.

#### Implementation of SBS-PA intervention

The *TFIR* DVDs were implemented during the first 10 min of the designated 30-min time for gross motor play (in the classroom setting). To implement the *TFIR* protocol, classroom teachers and students followed along with the *TFIR* DVD on a portable DVD player (which was provided to each classroom by the study staff for the duration of the study). *TFIR* DVDs were intended to be viewed by the classroom teacher while the students watched and followed the teacher’s lead. After the completion of the 10-min *TFIR* DVD, the students were allowed to engage in their usual gross motor playtime activities (unstructured play) for the remaining 20 min. The simplicity of the *TFIR* protocol enhanced teachers’ ability to implement the intervention. The *TFIR* protocol was implemented during the morning and afternoon designated gross motor playtime. Two *TFIR* DVDs (one for morning and one for afternoon playtime) were distributed to classroom teachers each week to ensure that preschool centers were implementing the same *TFIR* DVD during the intervention times. Each week, teachers were instructed to implement the assigned morning and afternoon DVD at the respective gross motor playtime. Each set of *TFIR* DVDs with its accompanying picture and verbal movement guide were dropped off the Friday preceding their implementation to give classroom teachers time to become familiar with the routine for following week. The previous week’s *TFIR* DVDs were picked up on Monday. Throughout the 6-month study, each morning and afternoon set of *TFIR* DVDs were repeated every eight weeks for a total viewing of three times.

### Control condition

The control intervention (UPA) consisted of traditional long bouts (30 min) of gross motor playtime and included supervised free time (unstructured) for the preschoolers to play on their own or with other children. During the gross motor playtime (either indoors or outdoors), children were provided with their usual play equipment, which was the current standard in the participating preschools. Children were allowed to choose from a variety of activities like running and jumping, bouncing, catching, and playing on structures or obstacle courses.

### Teacher training and process evaluation

To enhance the fidelity to the intervention, within each preschool center all classroom teachers participated in a two hour in-service training session. The training sessions were held separately for staff at each preschool center. Training for teachers assigned to the SBS-PA intervention, included information on the importance of PA and how to implement the *TFIR* protocol. During the training session, teachers were taught how to lead preschoolers in *TFIR* activities and were guided through three different *TFIR* routines. Training of the teachers from the control centers focused on the importance of allowing their students to play freely during the allocated gross motor playtime. All participating teachers were expected to follow their assigned protocols during designated gross motor playtime within each normal preschool day throughout the study. Research staff members were available throughout the 6-month protocol to assist classroom teachers in implementing their assigned intervention. At least once each week, research staff observed each participating classroom to determine if both the SBS-PA and UPA interventions were being implemented as designed.

### Assessments and measures

Participants total preschool day PA was assessed at baseline, and at 3-months and 6-months post-initiation of the intervention with the Actigraph accelerometer (GT1M, Actigraph, LLC, Pensacola, FL), which has been previously validated and used in preschool-age children [4,24]. The accelerometer was attached to an adjustable elastic belt and worn around participants’ waists at the center of their lower back to be unobtrusive [25]. We asked that children wear the accelerometers during the preschool day from Monday thru Friday during each assessment time point. Accelerometers were placed on children upon their arrival at their preschool center and removed before departing for home. Classroom teachers were instructed on monitor placement and asked to ensure accurate repositioning of the monitor whenever removed. The monitor was programmed to store data at 15-s epochs daily. To reduce the baseline PA accelerometer data, a custom software program was used to process all data using the Sirard et al. 15-s epoch count cut off [26]. The age-specific, 15-s counts cut-offs for 3, 4, and 5 year olds for the different activity intensities were sedentary ≤301, ≤363, ≤398; light 302–614, 364–811, 399–890; moderate 615–1230, 812–1234, 891–1254; and vigorous ≥1231, ≥1235, ≥1255, respectively [27]. For the study population, the preschool-day was defined as 7:00 am – 4:30 pm. Data from the 15-s counts cut-off were converted to average counts/min and the percent of time spent in the various activity intensity thresholds and used for analysis.

The Observational System for Recording Physical Activity in Children-Preschool Version (OSRAC-P) [28,29] was used to collect information about participant PA during gross motor playtime and the contextual circumstances (location of PA, group composition, and activity initiator) of their PA within their preschool environment. Direct observation of gross motor playtime PA was conducted on one day (in the morning and afternoon) during each assessment time point. Within each preschool center, two randomly selected classrooms were observed. Prior to the beginning of the data collection session, researchers randomly selected eight children (six to be observed, two alternates) to observe over the course of the gross motor playtime session. Children were observed for 15-s intervals and then, during the next 15 s, recorded one code for each of the four variables of interest (PA level, location of PA, initiator of PA, and group composition). Each child was observed for one, five-minute session, with the observer rotating which child was assess every five minutes. An alternate child was used only in the event that the child who was being observed left the observation area. Observational data were collected on HP IPAQ handheld computers and later downloaded into a database and analyzed. All observers underwent training and were required to demonstrate high inter-observer reliability prior to assessing PA using OSRAC-P. Five to 10% of all sessions were analyzed to assess inter-observer reliability.

A self-report questionnaire, completed by parents/guardians, was used to obtain each child’s demographic and SES data. Standing height to the nearest millimeter (direct reading stadiometer) and body weight to the nearest 0.1 kg (digital scale) were assessed with participants wearing light clothing, with shoes removed. Body mass index (BMI) was calculated as the weight in kilograms divided by the square of the height in meters. Parents/guardians reported the amount of time their child engaged in sedentary activity (e.g., watching television, playing video games, art work or crafts) using a self-reported survey [4,30,31]. Anthropometric measures and sedentary behavior (via questionnaire) were assessed at baseline and 6-month after the initiation of the intervention.

### Statistical analysis

The primary objective of the STEP study is to examine the effects of short bouts of structured PA to increase preschool-age children’s total school-day PA, as well as percent time spent in MVPA during the school day. The preschool center is the unit of randomization and intervention; the individual child is the unit of measurement. The analyses will use the child-level data, accounting for correlation of child responses within class and of classrooms within a center. For all analyses of primary outcomes and mediating variables we will use generalized linear modeling statistical methods. The methods allow assessment of a simple treatment comparison as well as multivariable modeling. Mediator and modifier significance (set *a priori* at a significance level of 0.25 or below) will be assessed univariately to determine which variables will be eligible for inclusion in the multivariable model. Intervention effects (change in PA) will be assessed with repeated measures mixed model analysis of variance or covariance, with childcare center as a random effect given that we do not expect that the within-group time trends will be homogeneous. Using a mixed effects model allows us to incorporate random effects for the center and class within center (clusters) and thereby model the correct covariance structure of the data and produce more accurate estimates of the effects under study and their standard errors. The distributions of individual variables will be evaluated and the data transformed as necessary to meet the assumptions of normality and homoskedasticity. For categorically scaled measures, we will use the generalized estimating equation methods for logistic regression analysis. Demographic variables will be added to multivariate models along with potential interactions with the intervention. Significant (at the p = 0.05 level) variables and interactions will be retained in the final model. For the baseline characteristics statistical comparison between groups was made with Wilcox Rank Sum tests for scaled variables and Chi-Square tests for categorical variables, with a two-sided alpha = 0.05. All analyses will be computed using SAS (version 9.2).

#### Sample size and power calculations

Sample size was determined using methods for studies with randomization by cluster [32]. With 25 children per preschool center and five preschool centers per condition, we have 80% power to detect a significant difference in the mean change in number of minutes of PA (2-sided) between the two conditions of between 5.5 and 10.7 min of total school day PA at the 5% significance level, depending on the correlation ranges above. Thus we have adequate power to detect a difference that would be significant both statistically and clinically. Based on the assumption that repeat measures of PA within clusters will only be moderately related after adjusting for repeat measures at the individual level, this power will hold similarly when analyzing the full (3 observation points) study using a random coefficients model [33].

## Results

This study is ongoing. We successfully recruited five preschool centers per treatment group (total of 10 preschool centers, 34 classrooms). A total of 680 students from all preschool centers were eligible to participate in the study. Of those, 331 families responded to our advertisement and were eligible for participation. Prior to the initiation of the study, 16 participants were terminated from the preschools, for a total of 315 children enrolled at baseline. Baseline assessment was completed by 92% of eligible families. Baseline variables did not significantly differ between preschool centers for each group (SBS-PA and UPA); therefore their data were combined for this report. Baseline socio-demographic characteristics are presented in Table 1. The study population consisted of 50.2% boys and 49.8% girls. The majority of the participants were either African-American (25%) or Latino/Hispanic (41%). The racial/ethnic composition of the participants is representative of the 10 preschool centers student population. Fifty-four percent of the study population was from single-parent homes and 59% of the study sample had annual household incomes less than $40,000.

**Table 1:** Baseline demographic characteristics

**Variable**	**SBS-PA**	**UPA**	**p**	**All**
	**(n = 141)**	**(n = 150)**		**(n = 291)**
Gender, n (%)				
Boys	59 (52%)	62 (49%)	0.65	121 (50.2%)
Girls	55 (48%)	65 (51%)		120 (49.8%)
Race/Ethnicity, n (%)				
White	36 (34%)	36 (29%)	0.13	72 (31%)
Hispanic	35 (33%)	59 (48%)		94 (41%)
African-American	33 (31%)	25 (20%)		58 (25%)
Asian	2 (2%)	3 (2%)		5 (2%)
Other	0 (0%)	1 (1%)		1 (0.4%)
Parent/guardian age (yrs)	43.8 ± 7.7	44.0 ± 7.6	0.73	43.9 ± 7.6
Parent/guardian BMI (kg/m^2^)	27.8 ± 6.1	27.4 ± 6.0	0.43	27.6 ± 6.0
Parent/guardian marital status, n (%)				
Married	51 (45%)	57 (45%)	0.27	108 (45%)
Divorced/Separated or widowed	12 (10%)	6 (5%)		18 (7%)
Single-never married	51 (45%)	63 (50%)		114 (47%)
Maximum household education level, n (%)				
High school graduate or less	31 (32%)	44 (36%)	0.64	75 (34%)
Some college/technical school	24 (24%)	32 (26%)		56 (25%)
College graduate	43 (44%)	46 (38%)		89 (40%)
Annual total household income, n (%)				
Less than $20,000	28 (26%)	34 (29%)	0.57	62 (27%)
$20,000 - $39,000	32 (29%)	40 (34%)		72 (32%)
$40,000 - $59,000	13 (12%)	15 (13%)		28 (12%)
Greater than $60,000	36 (33%)	29 (25%)		65 (29%)

Unless otherwise noted, variables are presented as mean ± sd. SBS-PA = Short bouts structured-physical activity; UPA = Unstructured physical activity; BMI = body mass index.

Baseline characteristics of the sample are presented in Table 2. The average age (mean ± SD) was 4.0 ± 0.0 for boys and 4.1 ± 1.0 for girls. Age and BMI (kg/m^2^) did not differ significantly by group. The average BMI for boys and girls were at the 69th and 70th age- and gender-specific percentiles, respectively. Significant between group differences were observed for BMI percentile (SBS-PA, 64.8 ± 28.4; UPA, 71.9 ± 24.5) The baseline prevalence of overweight (BMI ≥85th but <95th percentile) and obesity (BMI ≥95th percentile) in the SBS-PA and UPA groups were 19%, 12%, 22%, and 18%, respectively. No significant between group differences were observed for any PA variables. Participants in both groups spent 74.2% of their preschool day engaged in sedentary activity. Only, 6.4% of the preschool day (~9 h per day) was spent engaged in MVPA. During gross motor playtime, participants engaged in sedentary activity, light activity, and MVPA for 24.5%, 40%, and 33.6% of the observation intervals, respectively. There was a significant difference between the intervention and control groups for the observed intervals of light activity.

**Table 2:** Baseline and During Preschool Physical Activity Measures

**Variable**	**SBS-PA**	**UPA**	**p**	**All**
	**(n = 141)**	**(n = 150)**		**(n = 291)**
Age (yrs)	4.0 ± 0.8	4.1 ± 0.9	0.36	4.1 ± 0.8
Weight (kg)	17.3 ± 3.0	18.1 ± 3.5	0.04	17.7 ± 3.3
Height (cm)	102.4 ± 6.9	103.0 ± 6.3	0.39	102.7 ± 6.6
Body mass index (kg/m^2^)	16.4 ± 1.4	17.0 ± 2.2	0.06	16.7 ± 1.9
Body mass index percentile	64.8 ± 28.4	71.9 ± 24.5	0.03	68.5 ± 26.7
Screen media use (weekly minutes)				
Television viewing	87.9 ± 155.9	96.6 ± 165.7	0.81	92.5 ± 160.9
VCR/DVD	40.7 ± 113.1	42.2 ± 111.3	0.98	41.5 ± 111.9
Video game	13.3 ± 43.7	26.7 ± 77.1	0.12	20.4 ± 63.9
Percent time spent in Sedentary PA	75.4 ± 9.3	74.9 ± 9.4	0.18	74.2 ± 9.3
Percent time spent in Light PA	19.0 ± 4.8	18.1 ± 4.8	0.19	18.6 ± 4.8
Percent time spent in MVPA	6.7 ± 3.4	6.2 ±3.4	0.26	6.4 ± 3.4
Percent of direct observation at levels of PA				
Sedentary	26.9 ± 27.2	22.0 ± 25.6	0.26	24.5 ± 26.4
Light	31.2 ± 21.6	48.9 ± 23.4	0.0001	40.0 ± 24.1
MVPA	39.0 ± 31.0	27.5 ± 27.4	0.06	33.6 ± 29.8

Values are represented as means ± sd. SBS-PA = Short bouts structured-physical activity; UPA = Unstructured physical activity; PA = physical activity, MVPA = moderate to vigorous PA. Percent times spent in PA intensity are determined for the total preschool day. Percent of direct observation at levels of PA are determined for gross-motor playtime.

## Discussion and Conclusions

In the past two decades, the obesity rate of preschool-aged children in developed countries like the United States has increased at an alarming rate [34,35]. Because obesity tends to track from childhood into adulthood, experts have recommended that obesity prevention be initiated as early as possible, particularly in preschool-aged children [36,37]. PA and its counterpart, sedentary behavior, have been associated with the increased prevalence of obesity in children [37-40]. Therefore, effective interventions designed to increase preschool-aged children’s PA levels are desperately needed.

Approximately, 58% preschoolers in the US spend the majority of their day in an early childcare program (i.e., preschool centers) [41]. Therefore, early childcare programs present a unique opportunity to increase PA levels in this population. Due to the significant amount to time that preschoolers spend at the preschool setting, some states have started regulating PA policies. For example, in 2010, Massachusetts State Department of Early Education & Care mandated that preschool centers should provide a minimum of 60 min of daily gross motor playtime. Most preschools divide the 60-min block into two 30-min time blocks (one in the morning and one in the afternoon) of free unstructured playtime. Unfortunately, unstructured free playtime has been shown to have very little impact on preschooler’s PA levels [4]. In addition, researchers have shown that preschoolers tend to accumulate only 8–12 min of MVPA during a typical 30-min gross motor playtime [6,7].

In order to change the PA policy in preschoolers to increase the amount of MVPA in which they engage, changes are needed not only in the types of PA to which preschoolers are exposed, but also in the academic classroom. The STEP study examines the effects of short bouts of structured PA implemented within the classroom setting as part of designated gross motor playtime on during preschool total PA and sedentary time in preschoolers. We hypothesized that short bouts of PA just prior to engaging in outdoor activity can not only increase classroom PA, but can also increase the amount of MVPA in which children engage during unstructured playtime.

Of the 15 sites approached to participate in the program, we were able to successfully recruit 10 low-SES preschool centers. The established partnership with each preschool center allowed us to engage classroom teachers’ assistance in the recruitment of individual participants for the assessment portion of the study. The randomization process produced two groups with similar characteristics and only a few statistically significant differences. The number of significant differences identified between intervention and control groups in the current study could happen by chance. Most of the participants in the sample are of normal weight, however approximately 35% were classified as overweight/obese. The prevalence of overweight/obese status among preschool-aged children in the study sample is substantially higher than the reported prevalence (21.2%) in the 2007–2008 NHANES [34]. We observed a statistically significant difference in body weight and BMI percentile values between the two groups, with the UPA group being heavier than participants in the SBS-PA group. Although significant, the absolute numbers between the two groups was small. The significant difference in these values will be taken into account in analyzing the effect of the SBS-PA intervention on PA due to the correlation between weight and PA. Researchers have reported that overweight/obese children tend to be less active than their normal weight/leaner counterparts [42-44].

On average the participants in this study spent a large percentage of the preschool day in sedentary activity and only 6% of the time engaged in MVPA. Their reported baseline MVPA (accelerometer) level is similar to what others have reported in preschool-age children [4,29,45]. OSRAC-P (direct observation system) was used to assess participants’ PA during gross motor playtime. We observed that during this time period, participants engaged in MVPA approximately 33% of the observed intervals, similar to what other researchers have reported (27-40%) [6,7]. During the observed intervals, a significant between group difference was observed for light intensity activity. It is difficult to compare the PA data from accelerometery to that from direct observation because accelerometer data were collected for the entire preschool day and the direct observation data were only collected during gross motor playtime. To date classroom teachers have successfully implemented the *TFIR* DVDs. These teachers who are usually pressed for time have reported an increased ability to use the *TFIR* DVDs to expose the children to more structured bouts of physical activity.

Very few studies have been successful at increasing PA during the school day in preschool-aged children. Several states are passing non-funded regulatory PA policy mandating that preschool centers should provide a minimum of 60 min of daily gross motor playtime for preschool-aged children. However, most of these policies do not indicate how this time should be allocated. Most preschools divide the 60-min block into two 30-min time blocks (one in the morning and one in the afternoon) of free playtime (unstructured). But this approach does not increase PA [4]. Researchers have observed that preschoolers spend an average of 27–40% of a 30-min outdoor playtime engaged in MVPA [6,7]. The accumulation of MVPA during gross motor playtime generally occurs during the first half of playtime and represents a small fraction of the amount of time that preschoolers engage in MVPA. Based on this evidence, a better PA policy would be to expose preschoolers to shorter bouts of structured PA throughout the preschool day.

## Abbreviations

BMI, Body mass index; EPAO, Environment and Policy Assessment and Observation Audit Tool; IR, Instant recess; PA, Physical activity; MVM, Meta-Volition Model; MVPA, Moderate-to-vigorous physical activity; SBS-PA, Short bouts of structured PA; SES, Socioeconomic status; STEP, Short bouTs of Exercise for Preschoolers; TFIR, Tutti Fruitti Instant Recess; UPA, Unstructured PA.

## Competing interests

The author(s) declare that they have no competing interests.

## Authors’ contributions

SA conceived of the study and acquired funding. SA, ON, AM, and SS participated in data acquisition. SA, AL, and SS conducted the data analyses, however all authors participated in data interpretation. All authors participated in the design of the study. All authors were directly involved in drafting and edition of the manuscript. All authors have read and approved the final manuscript.

## Pre-publication history

The pre-publication history for this paper can be accessed here:

http://www.biomedcentral.com/1471-2458/12/582/prepub
